# A Smartphone App Combining Global Positioning System Data and Ecological Momentary Assessment to Track Individual Food Environment Exposure, Food Purchases, and Food Consumption: Protocol for the Observational FoodTrack Study

**DOI:** 10.2196/15283

**Published:** 2020-01-28

**Authors:** Maartje P Poelman, Frank J van Lenthe, Simon Scheider, Carlijn BM Kamphuis

**Affiliations:** 1 Chair Group Consumption and Healthy Lifestyles Wageningen University and Research Wageningen Netherlands; 2 Department of Human Geography and Spatial Planning Utrecht University Utrecht Netherlands; 3 Department of Public Health Erasmus University Medical Center Rotterdam Rotterdam Netherlands; 4 Department of Interdisciplinary Social Science Utrecht University Utrecht Netherlands

**Keywords:** ecological momentary assessment, eating behavior, environmental exposure, mobile apps, smartphone, geographic information systems, food preferences, diet records

## Abstract

**Background:**

Our understanding of how food choices are affected by exposure to the food environment is limited, and there are important gaps in the literature. Recently developed smartphone-based technologies, including global positioning systems and ecological momentary assessment, enable these gaps to be filled.

**Objective:**

We present the FoodTrack study design and methods, as well as participants’ compliance with the study protocol and their experiences with the app. We propose future analyses of the data to examine individual food environmental exposure taking into account the accessible food environment and individual time constraints; to assess people’s food choices in relation to food environmental exposure; and to examine the moderating role of individual and contextual determinants of food purchases and consumption.

**Methods:**

We conducted a 7-day observational study among adults (25-45 years of age) living in urban areas in the Netherlands. Participants completed a baseline questionnaire, used an app (incorporating global positioning system tracking and ecological momentary assessment) for 7 days, and then completed a closing survey. The app automatically collected global positioning system tracking data, and participants uploaded information on all food purchases over the 7-day period into the app. Participants also answered questions on contextual or individual purchase-related determinants directly after each purchase. During the final 3 days of the study, the participants also uploaded data on fruit, vegetable, and snack consumption and answered similar ecological momentary assessment questions after each intake.

**Results:**

In total, 140 participants completed the study. More than half of the participants said they liked the app (81/140, 57.9%) and found it easy to use (75/140, 53.6%). Of the 140 participants, 126 (90.0%) said that they had collected data on all or almost all purchases and intakes during the 7-day period. Most found the additional ecological momentary assessment questions “easy to answer” (113/140, 80.7%) with “no effort” (99/140, 70.7%). Of 106 participants who explored their trips in the app, 20 (18.8%) had trouble with their smartphone’s global positioning system tracking function. Therefore, we will not be able to include all participants in some of the proposed analyses, as we lack these data. We are analyzing data from the first study aim and we expect to publish the results in the spring of 2020.

**Conclusions:**

Participants perceived the FoodTrack app as a user-friendly tool. The app is particularly useful for observational studies that aim to gain insight into daily food environment exposure and food choices. Further analyses of the FoodTrack study data will provide novel insights into individual food environmental exposure, evidence on the individual food environment-diet interaction, and insights into the underlying individual and contextual mechanisms of food purchases and consumption.

**International Registered Report Identifier (IRRID):**

DERR1-10.2196/15283

## Introduction

### Background

The prevalence of obesity- and nutrition-related noncommunicable diseases, such as type 2 diabetes and cardiovascular diseases, has increased substantially in recent decades [1]. Multiple factors contribute to these increased prevalences, although the changed food environment is regarded as an important driver [2]. Although the associations between temporal food environmental changes and diet and obesity have been described extensively [2-4], a better fundamental understanding is needed of how and where people purchase and eat food in contemporary obesogenic environments.

Many studies have focused on the community food environment, operationalized as the number, type, location, or accessibility of food outlets [5]. This is often expressed for a certain area—often the residential food environment (eg, supermarket or fast-food outlet availability within 1 km of the home address)—and how this fixed environment affects food choices [6] or health [7]. However, there are several limitations when studying only associations between the residential community food environment and food choices.

First, a person’s food environment is not limited to the residential area [8,9]. People undertake several activities during the day (eg, going to work, shopping, and participating in recreational activities) that usually require them to travel beyond their residential area. The compilation of all visited places, and the trips in between, should therefore be the basis on which to define a person’s daily food environmental exposure. This notion has been more frequently acknowledged in recent years, and researchers have used diverse spatiotemporal approaches and statistical methods assessing daily activity spaces to define the food environmental exposure [10-12]. Although such activity space–based food environments provide a more realistic picture of an individual’s total exposure than the picture obtained by focusing only on what is available in the residential neighborhood, action spaces do not encompass the totality of people’s accessible food environment [13]. Thus, the calculation of food environment exposure based on activity spaces ignores the food outlets that individuals potentially would have been able to visit because they were reachable within the time-space window available for food shopping at that point in time, but that were not on the individual’s route. In addition, such calculations are affected by selective daily mobility bias. For example, when an activity (eg, going out to eat) influences both the place visited (eg, fast-food outlet) and the activity practiced (eg, eating fast food), the spatial access to environmental resources is calculated based on these intentionally visited places (which participants would not have been exposed to had they not intended to conduct the activity) [14]. Activity spaces do not account for this bias and therefore may result in overestimation of the association between the activity space–based fast-food environmental exposure and fast-food consumption.

Second, food environmental exposure is most often studied in association with dietary intake, and not with food purchases. However, food purchase behavior is conceptually more directly linked than dietary intake is to food environment exposure (although some products are directly consumed at the outlet where they are purchased), and including both eating and purchasing behavior will best enable an understanding of the link between the environment and obesity. So far, only a few studies have focused on food purchases in association with food environmental exposure [15-17].

Third, studies assessing the environment-diet relationship often ignore the perspective of time, defined as “time of the day” or “individual time perspectives.” We do not yet understand the role of time in the environment-diet relationship. On the sociobehavioral level, time pressure is reported as a barrier to eating a healthy diet [18], influenced by demands such as employment or childcare responsibilities [19]. People’s use of or susceptibility to the food environment may differ over the time of day. In addition, concepts from time geography [20], in particular space-time paths and space-time prisms, could play an important role in research on time constraints. A space-time path identifies stable daily routines, such as work, home, and leisure activities, in space and time. A space-time prism identifies the places that are within reach of a person within a given time window. Both concepts can be used in geographic information systems (GISs) to make time-dependent assessments [21] of both spatial food habits and alternative behavior under temporal constraints.

Fourth, the mechanisms through which the daily food environment affects food choices (ie, food purchases and consumption) are still unknown. Individual and contextual factors may interfere in the environment-diet relationship. For example, individuals under stress may have less control over food choices and are more susceptible to unhealthy food consumption [22,23].

Novel smart technologies for data collection allow the aforementioned limitations and gaps in the literature to be addressed and will improve the understanding of the role of day-to-day food environments in food choices. The FoodTrack study uses a specially developed smartphone app for its main measurements. It includes a novel interface to report food purchases and consumption, and incorporates such technologies as a global positioning system (GPS) and ecological momentary assessment (EMA).

### Objectives

The FoodTrack study has four aims: to examine individual food environmental exposure in a fine-grained manner (aim 1), taking into account the accessible food environment and individual time constraints; to assess people’s food choices (purchases and consumption) in relation to food environmental exposure (aims 2 and 3); and to examine the moderating role of individual and contextual determinants (eg, mood, companion, time of the day) of food purchases and consumption (aim 4).

Here, we present the protocol and study design of the FoodTrack study, and describe the smartphone app. We also discuss the participants’ compliance with the study protocol and their experiences with the FoodTrack smartphone app.

## Methods

### Design and Setting

We used a smartphone app incorporating GPS tracking and EMA during a 7-day observational study to collect data from adults (25-45 years old) living in urban areas in the Netherlands. We set the minimum age at 25 years to exclude those undertaking full-time studies at an educational or training institute; as such, study participants were not representative of the country’s population. The study did, however, include the majority of generation Y (millennials) and the youngest wave of generation X, whom we included in the study because we had hypothesized that during their daily lives, these people juggle many activities (eg, work, caregiving, leisure activities) that could interfere with health behaviors (such as food choices). The study was funded by the Netherlands Organization for Scientific Research and approved by the ethical committee of the Faculty of Social Sciences of Utrecht University, the Netherlands (FETC18-014).

### Recruitment and Study Procedure

Between March and July 2018, we recruited participants via online platforms (eg, Facebook, news websites) and through advertisements in national and local newspapers. Additional interest in the study was generated through interviews with the involved researchers about the FoodTrack study broadcast by national and local radio stations. Adults who were willing to participate were invited to visit the FoodTrack website [24], where they could read more about the study, watch a short video clip about the study, or request more information via email, if needed. If they were interested, potential participants were asked to complete an online application form, which included questions related to the inclusion criteria. Those who met the inclusion criteria were invited to complete the online informed consent form and were then directed to the online baseline questionnaire (pretested on usability and technical functionality). Once they had completed it, they were sent an email confirming their initial participation and providing further details (eg, their log-in name and password, the start day of the research). The start day (Monday-Sunday) of the study was randomly assigned to each participant, so that data on fruit, vegetable, and snack consumption would be collected for both weekdays and weekends. Participants were sent a separate text message with a link to download the app, which was freely available from the App Store (Apple Inc, Cupertino, CA, USA) or Google Play (Google LLC, Mountain View, CA, USA). Once logged in, participants were asked to allow the app to access their location (thus allowing GPS tracking) and to receive push messages (ie, reminders). The study website provided an introductory video, as well as a video outlining the use and functionalities of the smartphone app. An online manual (in pdf format) explaining the use and functionalities of the app in more detail was also provided. Participants were able to contact the research team by calling or messaging us during the entire study period with any study-related questions or problems.

The smartphone app automatically collected GPS tracking data in the background. The participants used the app to upload information on all food purchases over the 7-day period and to answer EMA questions about contextual and individual purchase-related factors. These questions popped up whenever they uploaded data about a food purchase. Purchases were defined as food and drink products purchased for immediate consumption (eg, restaurant meals, snacks on the go), to be consumed later (eg, groceries, small packaged foods), or to also be consumed by others (eg, groceries for the entire household). In addition, during the final 3 days of the 7-day period, participants also collected data about the consumption of fruit, vegetables, and snacks (including fast foods) and answered EMA questions about contextual and individual consumption-related factors whenever they uploaded data about food they consumed. The participants were rewarded with an online non–food shopping voucher worth €30 (about US $33) if they completed all elements of the study. At the end of the 7-day period, they completed the online closing survey. The original questionnaires were in Dutch but we translated the responses into English for this paper.

### Inclusion Criteria

People were eligible to participate in the FoodTrack study if they self-reported that they met the following criteria: (1) living in an urban area in the Netherlands, (2) aged between 25 and 45 years, (3) not being a full-time student, (4) possessing a smartphone (iOS or Android device), and (5) not having 1 or more of the following conditions: physical disability influencing daily mobility (eg, wheelchair); prescribed or medical diet (by a medical doctor or dietitian); gastric bypass; eating disorder; or taking medication affecting appetite.

### The FoodTrack Smartphone App

#### Development

The FoodTrack smartphone app was developed for this study in collaboration with a commercial company, Locatienet [25]. It was based on an existing tracking app [26] with new elements—the registration tools for food purchases and consumption, and the EMA questions—built in. During the development phase, we pilot tested (n=20; unpublished data, 2017) and then further refined the app. For example, we added the ability to see an overview of the food purchases and consumptions already submitted. In addition, we added the time and date of purchase or consumption as an extra item to improve data quality, as well as a few extra answer options to improve the convenience of the app. The app’s details and functionalities are specified below. Multimedia Appendix 1 provides several screenshots.

#### Tracking Locations and Trips

The app ran in the background and used the smartphone’s sensing capabilities (eg, GPS, accelerometer) to detect, record, and quantify the movements of participants, the trips they made, and the places they visited [26]. We will use these data to calculate individual places visited and purchases made within the living environment (aim 1).

The app’s menu included a button labelled “see trips” that enabled participants to see the trips they had made and the locations they had visited during the day, including the date, time, and start and stop locations. They could also view each trip on a map by clicking on the trip concerned.

#### Tracking Food Purchases and Consumption

We asked the participants to upload their purchases as soon as possible after they had paid for them. They could either take a photo of the receipt listing the products bought along with their prices and their weights, or enter their purchases manually (product and amount purchased, eg, 1 kg of sugar; or predefined serving, eg, 1 candy bar, 35 g) by means of the integrated food database. This database was built upon the Web-based food diary Eetmeter of the Netherlands Nutrition Centre [27], which includes food products available in the food databank of the National Institute of Health and Environment and the Netherlands Nutrition Centre [28]. Although the databank includes many products, it does not include all products (ie, not all specific brands or types are present). Therefore, participants were instructed to enter the food product that most closely corresponded to what they ate if the specific product or brand type was not available (eg, when eating a Big Mac, they entered this as cheeseburger) and the number of items or the estimated serving size (in grams). Participants also recorded their consumption of fruit, vegetables, and snacks (including fast foods) by manually entering their consumption into the FoodTrack app. Participants were not asked to take photos of the food consumed.

After entering the types and amounts of food products they had purchased or consumed, the participants added information concerning when and where they had done so and answered short questions about the contextual and psychosocial determinants of the consumption, by means of the short EMA survey outlined below.

#### Ecological Momentary Assessment to Collect Contextual Factors of Food Purchases and Consumption

We integrated an EMA survey instrument into the smartphone app (using Survey Project [29]), allowing for the collection of data on food purchases, food consumption, and individual- or contextual-related food choice determinants (Multimedia Appendix 2). After participants had entered the food products purchased or consumed, short EMA questions concerning the following variables were provided.

#### Characteristics of the Purchase or Consumption

We assessed the type of purchase by asking whether the purchase was a “snack/drink” (“in between meals”) or “a meal” or “groceries.” We also asked participants whether the food purchase was for “themselves,” “for others,” or for “both themselves and others.”

To determine the type of consumption, we asked participants whether they consumed fruit, vegetables, or snacks “in between meals” or “as part of a meal.”

Participants selected from multiple response options to indicate whether their food purchase or consumption was spontaneous (impulsive), planned (a routine), or a combination of both.

#### Spatiotemporal Characteristics of the Purchase or Consumption

Participants reported information about the location of their purchase or consumption either immediately after the purchase or consumption by clicking on the “current location” button, or at a later point in time by entering the location data manually, although this was highly discouraged.

Participants selected from multiple response options (which included an open answer box) to indicate the type of food outlet or the location where they purchased or consumed the food.

Participants were asked to report the exact time and date of the food purchase or consumption. The current time and date were presented as the default.

#### Social Characteristics of the Purchase or Consumption

Participants selected from multiple response options (which included an open answer box) to indicate whether they had a companion or companions during the purchase or consumption event (eg, being alone, with family, with colleagues).

Participants selected from multiple response options (which included an open answer box) to indicate whether they were involved in an activity during the purchase or consumption event (eg, doing nothing else, shopping, working).

#### Individual Characteristics of the Food purchase or Consumption

Participants selected from multiple response options (“not at all,” “a little,” “somewhat,” “very much”) to indicate whether they felt “tired,” “stressed,” or “in a rush” during the purchase or consumption event.

#### Overview of the Food purchases or Consumption

On the app menu, participants could see either their registered food purchases or their registered food consumption. An overview of the registered products purchased or consumed was provided per day, including details concerning the type of purchase, type of outlet (eg, supermarket), location (address), and company (eg, family).

#### Reminders and Prompts

The day before the start of the study, participants received a push message saying (translated from Dutch) “Welcome to the study. Please register your food purchases as of tomorrow.” During the morning of the fifth day, participants also received the push message “From today onward, please also register what you consume.” During the rest of the study, participants received 2 reminders each day (at 2 PM and 8 PM), but only if they had not registered any purchases or consumption (“Did you purchase something today? Please register it.”). Participants were not able to customize the reminders.

In addition to the reminders sent automatically via the app, we sent preprogrammed reminder messages to keep participants engaged in the study and encourage them to ask questions if they had any difficulties with the data collection.

#### Settings

The app’s settings menu enabled participants to verify whether the Wi-Fi, GPS, and mobile network were functioning properly and to consult the Frequently Asked Questions section concerning the app and the study (eg, “How do I register vegetables?” “What are ‘snacks?”). Participants were also able to change their password via the settings and to use the menu to log out.

### Measures

#### Online Baseline and Closing Survey

We used the online baseline survey to assess sociodemographic characteristics and weight status, and to briefly screen healthy (ie, fruit) and unhealthy (ie, savory snacks) food products. We also measured psychosocial variables associated with eating behavior.

The closing survey assessed several additional food choice-related questions. Multimedia Appendix 2 shows a detailed overview of the measures included in the baseline and the closing surveys and item examples.

#### Food Environment: Geographic Information System Measures

To assess food environmental exposure, we will link the GPS tracks measured by the app with the retail food outlet database maintained by Locatus [30]. This database contains retail information independently sourced via annual onsite surveys, including data on 27 types of food outlets (eg, supermarkets, fast-food outlets, greengroceries, bakeries, shops selling fresh and fried fish, and restaurants).

#### Process Evaluation of Experiences With the App and Participants’ Compliance With the Study Protocol

We used 2 items to assess the app’s general ease of use and likeability. We also assessed whether the participants had noticed the messages that were sent automatically by the app to remind them to register data on food purchases and consumption, and whether these had helped to improve compliance (2 items). With respect to entering food choices in the app, we also used 2 items to assess the completeness of the food product database and how easy it had been for the participants to estimate and enter the portion sizes of the products purchased or consumed, and how accurate they had been.

We used 4 items to assess the use and operability of the app. We assessed whether participants had allowed it to track them throughout the research period, whether they had explored the trips that the app had tracked automatically, and whether they considered these tracks accurate. We also assessed whether they had experienced a dead battery during the research period.

We used 5 items to assess whether the participants had entered all their groceries and other purchases (eg, snacks on the go), and their intake of fruits, vegetables, and snacks. If they had deliberately not done so, we assessed the reasons for this. We also asked the participants whether they had entered the products immediately after purchase or consumption (as requested) or at a later time. Finally, we assessed how easy it had been to answer the additional questions with respect to individual or contextual factors related to the food choice event, and how much effort doing so had required. Finally, the participants could use an open question to provide any comments they wanted with respect to the app or the overall study.

### Data Analyses

In this paper, we provide insight into the descriptive statistics of the participants’ compliance with the study protocol, and their experiences with and evaluation of the smartphone app and the study. To assess food environmental exposure in future studies, we will assess individual space-time paths using individual GPS tracks to extract home and other important locations. Subsequently, we will assess the individuals’ accessible food environment, by extracting food events from GPS tracks and by computing corresponding space-time prisms using GISs to identify the location of food outlets that are within reach of a person. After assessing individual exposure, we will assess the association between individual food environmental exposure and food choices (purchases and consumption) using linear mixed-model analyses. We will assess effect modification by means of psychosocial variables (eg, “Is the association between environmental exposure and food purchases different for individuals who experience time pressure and those who do not?”).

## Results

### Participant Characteristics

**Figure 1:**
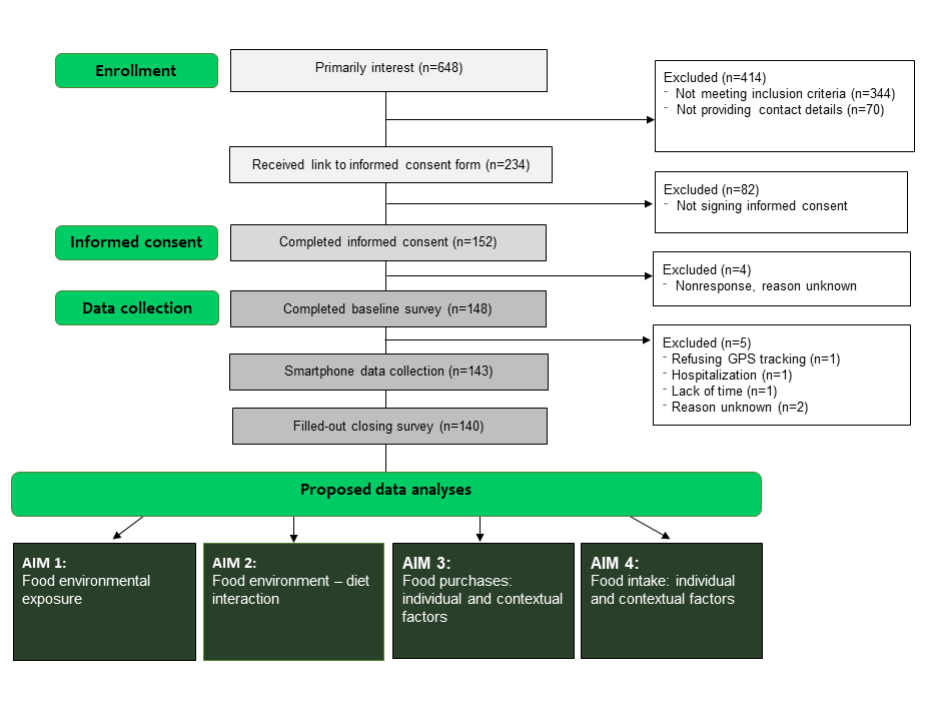
Flowchart of the FoodTrack study. GPS: global positioning system.

In total, we screened 648 people who expressed an interest in participating in the study. Of these, 304 (46.9%) met the inclusion criteria (n=344 did not, mainly because they were too young or too old, or were still studying). However, 70 of those who met the criteria did not provide their contact details (email, telephone number), and were therefore excluded from the study. We sent the remaining 234 potential participants a link to an online informed consent form; 152 signed the form and confirmed that they wished to participate. Of this latter group, 143 participants filled out the baseline survey and used the FoodTrack app; however, 3 of these participants did not complete the closing survey (Figure 1). Table 1 provides the sociodemographic characteristics of the 140 participants who completed the study. Of the 140 participants, 120 (85.7%) were women; the mean age was 33.8 (SD 6.36) years; 111 (79.8%) had attained a high educational level; 5 (3.6%) had a low educational level; 54 (38.6%) reported daily fruit intake; and 75 (53.6%) reported daily vegetable consumption.

**Table 1:** Characteristics of the participants who completed the entire study (N=140).

Characteristics		Mean (SD) or n (%)
Age (years), mean (SD)		32.8 (6.4)
Female sex, n (%)		120 (85.7)
Dutch origin, n (%)		131 (93.6)
**Marital status, n (%)**		
	Married or registered partnership	30 (21.5)
	Unmarried	95 (67.9)
	Divorced	5 (3.6)
	Other	10 (7.1)
**Household composition, n (%)**		
	2 adults, no children	44 (31.7)
	2 adults, with 1 or more children	29 (20.8)
	1 adult, with 1 or more children	6 (4.4)
	Single adult	51 (36.7)
	Living with others (parents, group)	6 (4.3)
	Other	3 (2.1)
**Educational level, n (%)**		
	Low	5 (3.6)
	Middle	23 (16.6)
	High	111 (79.8)
**Household income, in euros/month, n (%)**		
	<1500	14 (10.0)
	1500-2500	53 (37.9)
	>2500-4500	36 (25.7)
	>4500	25 (17.9)
	Not stated	12 (8.6)
**Food consumption behavior, n (%)**		
	Daily fruit consumption	54 (38.6)
	Daily vegetable consumption	75 (53.6)
	≤3 times/month sugar-sweetened beverage consumption	77 (55.0)
	≤3 times/month small snack consumption	15 (10.7)
	≤3 times/month large snack consumption	40 (28.6)

### Participant Experience of the App

Table 2 provides a detailed overview of the operability and use of the smartphone app. More than half of the 140 participants said they liked the app (n=81, 57.9%) and found it easy to use (n=75, 53.6%). However, the majority of the participants reported, for example, that they could not enter a specific brand, taste (eg, elderflower lemonade), or type of product (green pepper). In addition, composite meals or products (eg, a salad from the salad bar, or ready-to-eat meals) were mentioned as not being available in the app. Of the 140 participants, 21 (15.5%) had experienced a dead battery during the data collection period. Of 106 participants who explored the trips, 17 (16.0%) did not track all trips and 3 (2.8%) did not track any trips, which led to incomplete GPS tracking data collection.

**Table 2:** Process evaluation outcomes of FoodTrack app use (N=140 participants).

Response		n (%)
**Liked the app**		
	≤2 (negative)	14 (11.5)
	3 (neutral)	43 (30.7)
	≥4 (positive)	81 (57.8)
**App was convenient to use (easy to use)**		
	≤2 (negative)	29 (20.7)
	3 (neutral)	36 (25.7)
	≥4 (positive)	75 (53.6)
**Noticed the app reminders**		
	Yes	116 (82.9)
	No	24 (17.1)
**If noticed, reminders (n=116) evaluated as useful**		
	≤2 (not useful)	11 (9.5)
	3 (neutral)	25 (21.6)
	≥4 (useful)	80 (69.0)
**Food database complete**		
	Yes, all products	56 (40.0)
	No, not all products	84 (60.0)
**Ease of estimating the amount (portion size) of the purchased or consumed food**		
	≤2 (not useful)	49 (35.0)
	3 (neutral)	37 (26.4)
	≥4 (useful)	44 (38.6)
**Accuracy of participant’s estimate of food portion**		
	≤2 (not accurate)	14 (10.0)
	3 (neutral)	41 (29.3)
	≥4 (accurate)	85 (60.7)
**Allowed global positioning system for all days**		
	Yes	118 (84.3)
	No	14 (10.0)
	Don’t know	8 (5.7)
**Explored the trips**		
	Yes	106 (75.7)
	No	34 (24.3)
**If yes (n=106), the trips were accurate**		
	Yes, all	27 (25.5)
	Yes, most	55 (51.9)
	Yes, some	17 (16.0)
	No, trips were not registered	3 (2.8)
	Don’t know	4 (3.8)
**Experienced dead battery during day**		
	Yes	21 (15.0)
	No	114 (81.4)
	Don’t know	5 (3.6)

### Participant Compliance

Table 3 provides a detailed overview of the participants’ compliance with the study protocol during the data collection period. Of the 140 participants, 126 (90.0%) reported that they had collected data on all or almost all purchases and intakes during the study period. The 14 (10.0%) participants who had deliberately not entered all products had various reasons for this, including “product is unhealthy” (n=3) and “too much effort/complicated” (n=4). Moreover, of the 140 participants, 50 (35.7%) had entered most or all of their food purchases and 56 (40.0%) had entered most or all of their consumption more than 15 minutes after the event, although we had explicitly encouraged them to enter the data within 15 minutes. The majority of the participants found the additional EMA questions “easy to answer” (n=113, 80.7%) with “no effort.” (n=99, 70.0%).

Of the 140 participants, 66 (47.1%) answered the open question. They mentioned, for example, not only that it had been “fun to participate” or it was a “nice study” (n=13), but also that they had experienced struggles during the study: “not all products were available in the database” (n=11), “trips were not registered” (n=7), it was not possible to navigate back and forth in the app (n=12), and the “app was slow” (n=16).

With respect to the entire study project, we are analyzing data concerning the first project aim and expect to publish the results in the spring of 2020. We plan to publish further papers providing insights into the additional aims in the next 2 years as well.

**Table 3:** Process evaluation outcomes of data collection using the FoodTrack app (N=140 participants).

Response		n (%)
**Entered groceries**		
	Yes, all of my groceries	109 (77.9)
	Yes, most of my groceries	17 (12.1)
	Yes, a few groceries	5 (3.6)
	No, I did not enter my groceries	3 (2.1)
	No, but I did not do grocery shopping	6 (4.3)
**Entered all other food purchases**		
	Yes, all of my other food purchases	98 (70.0)
	Yes, most of my other food purchases	28 (20.0)
	Yes, a few other food purchases	6 (4.3)
	No, I did not enter my other food purchases	1 (0.7)
	No, but I did not purchase any other food	7 (5.0)
**Entered vegetables consumed**		
	Yes, all of the vegetables consumed	74 (52.9)
	Yes, most of the vegetables consumed	34 (24.3)
	Yes, some of the vegetables consumed	7 (5.0)
	No, I did not enter the vegetables consumed	20 (14.3)
	No, but I did not eat any vegetables	5 (3.6)
**Entered fruits consumed**		
	Yes, all of the fruits consumed	99 (70.7)
	Yes, most of the fruits consumed	19 (13.6)
	Yes, some of the fruits consumed	3 (2.1)
	No, I did not enter fruit consumed	9 (6.4)
	No, but I did not eat any fruits	10 (7.1)
**Entered snacks consumed**		
	Yes, all of the snacks consumed	87 (62.1)
	Yes, most of the snacks consumed	34 (24.3)
	Yes, some of the snacks consumed	7 (5.0)
	No, I did not enter any snacks consumed	7 (5.0)
	No, but I did not eat any snacks	5 (3.6)
**Time of entering purchase in app**		
	All purchases entered immediately after purchase (<15 min)	13 (9.3)
	Most purchases entered immediately after purchase (<15 min)	72 (51.4)
	Most purchases entered at later point in time (>15 min)	38 (27.1)
	All purchases entered at later point in time (>15 min)	12 (8.6)
	Did not purchase anything	5 (3.6)
**Time of entering consumption of snacks, fruit, or vegetables in app**		
	All foods consumed entered immediately after consumption (<15 min)	8 (5.7)
	Most foods consumed entered immediately after consumption (<15 min)	72 (51.4)
	Most foods consumed entered at later point in time (>15 min)	31 (22.1)
	All foods consumed entered at later point in time (>15 min)	25 (17.9)
	Did not consume snacks, fruit, or vegetables	4 (2.9)
Deliberately not entered a product		14 (10.0)
**Reasons (of n=14) for not entering a product**		
	Product unhealthy	3 (21.4)
	Too much effort, complicated	4 (28.8)
	Product I normally do not buy or consume	1 (7.1)
	Did not find the app user-friendly	1 (7.1)
	Small intake, so negligible	1 (7.1)
	No internet	1 (7.1)
	Could not find the product (or alternative)	2 (14.3)
	Product was for someone else	1 (7.1)
**Ease or difficulty of answering the ecological momentary assessment questions**		
	<2 (negative) = difficult	9 (6.4)
	3 (neutral) = neutral	18 (12.9)
	>4 (positive) = easy	113 (80.7)
**Effort to answer the ecological momentary assessment questions**		
	<2 (negative) = a lot of effort	19 (13.5)
	3 (neutral) = neutral	23 (16.5)
	>4 (positive) = no effort	99 (70.0)

## Discussion

### Principal Findings

This paper outlines the protocol of the FoodTrack study, as well as participants’ compliance with the study protocol and their experiences during study participation. We collected data over a 7-day period by means of the FoodTrack app, which uses GPS tracking and EMA to assess real-time individual and contextual variables at the moment of purchase or intake. The GPS tracking will allow us to assess participant activity-travel patterns that in turn will allow us to assess individuals’ daily food environmental exposure. The results of this study indicated that participants liked the app and found it easy to use. About 90% of the participants reported that they had collected data on all or almost all purchases and intakes during the 7-day period. However, not all participants complied with the study protocol that encouraged them to enter purchases or consumption right away. Nevertheless, the compliance figures of our study are in line with previous studies that used EMA in diet-related studies [31]. Qualitative insights resulting from the process evaluation also indicated that a minority of the participants encountered a few problems when using the app (eg, the app was too slow, not all products were present). We should carefully consider the likely implications of this when interpreting the results of the proposed studies. The outcomes of our process evaluation, however, provided us with information concerning which participants to include in, and which to exclude from, the proposed analyses. For example, we will exclude from the analyses participants who reported not collecting data on their grocery (n=3) or other purchases (n=1). Although this will decrease power, it will provide us with a more accurate estimation of food purchases in relation to food environmental exposure.

### Lessons Learned

To the best of our knowledge, this is one of the first studies that measured not only food outlet exposure and food outlet choice, but also actual food choice behavior within these outlets, as well as related individual and contextual factors, using smartphone technology measurements. This will provide more detailed insights into the food environment-diet relationship. During several phases of our study (eg, development of the app, preparation of the data collection, the process evaluation), we learned some lessons that may help researchers in this field to improve their future studies.

First, the study and the FoodTrack app were designed and implemented by an interdisciplinary team of colleagues from public health and nutrition and from transport and urban geography backgrounds. This interdisciplinary approach provided us with the opportunity to use geostatistical programs to conduct the proposed analyses to assess food environmental exposure. We highly advise nutrition or health scientists (or scientists from similar fields) planning to conduct a similar study using GPS tracking to collaborate closely with GIS experts in the development of their study and especially when analyzing the collected GPS data.

Second, the development of the smartphone app took a considerable amount of time (eg, finding an affordable and reliable party to collaborate with, conducting the app design phases, improving app functionality). Those intending to develop such an app should reckon on a lengthy development process (of, for example, at least 6-12 months). Although we pilot tested and refined the FoodTrack app in the development phase, some participants had trouble operating the app, especially concerning GPS tracking. Therefore, we will not be able to include all participants in some of the proposed analyses, as we lack their GPS data. Future studies can take account of this by recruiting participants with more advanced smartphones (eg, with better accelerometers) or by complementing smartphones with additional GPS trackers.

Third, despite our efforts to recruit a wide range of participants (eg, through using plain language in recruitment, being visible on social media, participating in interviews by local radio stations, and using audiovisual messages to recruit, such as video clips), the majority of participants were women and highly educated. This is not a representative sample of 25- to 45-year-old adults living in urban areas in the Netherlands [32]. Moreover, we requested that potential participants possess a smartphone, but in 2017, 9% of the adult population of the Netherlands did not own a smartphone [33], which could have led to selection bias. Although it is not the primary aim of the study, this makes it difficult to assess socioeconomic differences in the environment-diet association, due to small study samples. Researchers planning similar studies to assess socioeconomic inequalities in the environment-diet relationship should address this issue by employing recruitment strategies that appeal to a wider range of participants (eg, by recruiting participants face-to-face). Moreover, future studies could lend smartphones to participants who do not possess one.

Fourth, a small number of participants indicated that they had deliberately not entered their purchases or consumption, especially of vegetables. A likely explanation is that they found it difficult to enter vegetables as single products when they were components of a composite meal (eg, the tomatoes in spaghetti bolognaise). To improve compliance, future studies could optimize the app by allowing participants to take a photo of their food and simply describe the vegetables included in the product [34]. Future research using the FoodTrack app could improve it by optimizing GPS tracking, allowing customized settings (eg, prompts), or integrating additional strategies (eg, gamification strategies [35]) to improve usage.

### Strengths and Limitations

An important strength of the FoodTrack study is that we will be able to link collected GPS data and individual sourced data to GIS data on food outlet locations, in order to estimate individuals’ daily food environment exposure. A limitation of the FoodTrack study is that we relied on self-reported data regarding food purchases and consumption (and psychosocial and contextual variables). In addition, only 3 days of data on fruit, vegetable, and snack consumption were collected, precluding an insight into weekly consumption habits. As emphasized by previous research on dietary assessments [36], and confirmed by our process evaluation, it is clear that the collected data on food purchases and consumption should be included in the analyses in a rather qualitative way (eg, number of items purchased), rather than trying to assess, for example, the exact amount purchased or consumed or the daily nutrients obtained. The process evaluation has provided us with insight into participants’ use of the app during the data collection period. For example, some participants did not collect data on their food purchases or consumption during their participation. Although we still rely on self-reported insights, these data will help us to select participants who did comply with the study protocol.

### Conclusion

This study protocol provides insights into the design of the FoodTrack study and its procedures. It also provides detailed insights into the practical and operational issues involved in performing the study, including the participants’ use of and experience with the FoodTrack app. The FoodTrack study will add to our understanding of the role of day-to-day food environments and contextual factors in food choices, and will provide novel insights into individual food environmental exposure, innovative evidence on food environment-diet relations, and insights into the interplay with the individual and contextual mechanisms involved in food purchases and consumption.

## Appendix

Multimedia Appendix 1 Screenshots of the FoodTrack app.

Multimedia Appendix 2 Overview of the baseline and closing surveys of the FoodTrack study.

## Abbreviations

EMA: ecological momentary assessment
GIS: geographic information system
GPS: global positioning system
