# Supplementary figures and images for: A Smartphone App Combining Global Positioning System Data and Ecological Momentary Assessment to Track Individual Food Environment Exposure, Food Purchases, and Food Consumption: Protocol for the Observational FoodTrack Study
Source: JMIR Res Protoc. 2020 Jan 28;9(1):e15283. doi: 10.2196/15283 (PMC7013628; doi:10.2196/15283)

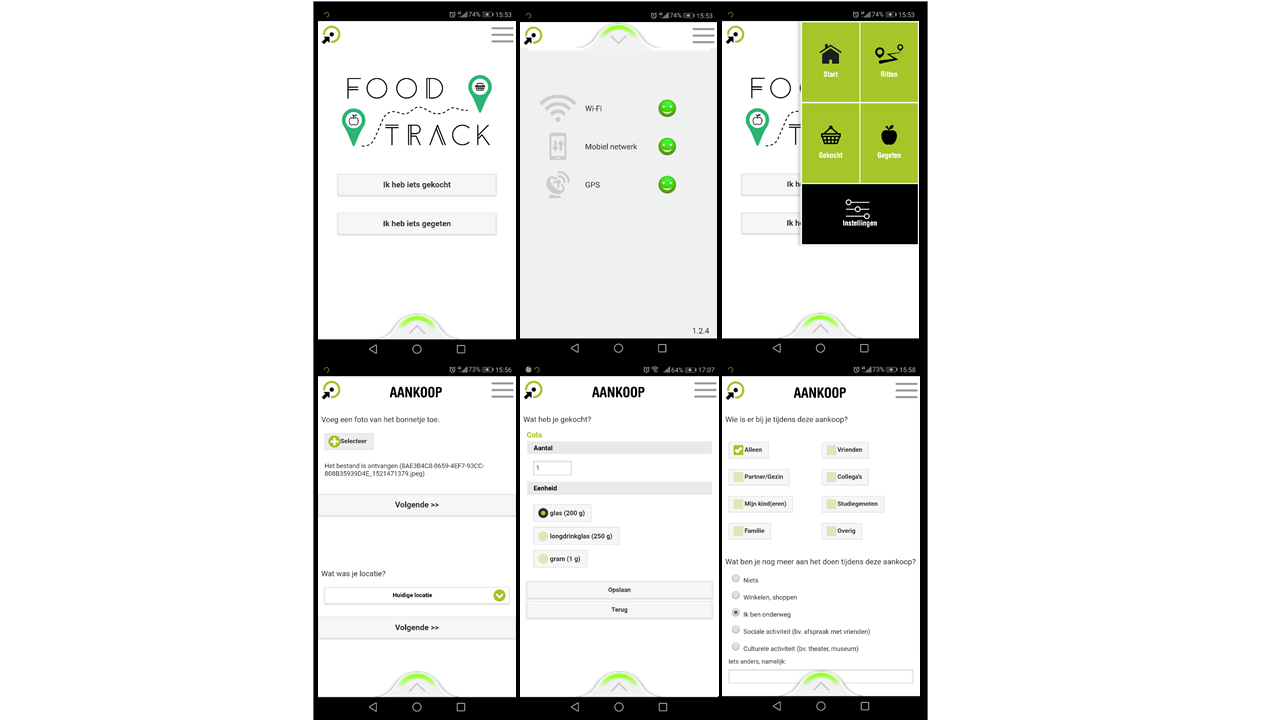

Supplement: Multimedia Appendix 1 [file resprot_v9i1e15283_app1.png]
