# Supplementary material for: A Smartphone App Combining Global Positioning System Data and Ecological Momentary Assessment to Track Individual Food Environment Exposure, Food Purchases, and Food Consumption: Protocol for the Observational FoodTrack Study
Source: JMIR Res Protoc. 2020 Jan 28;9(1):e15283. doi: 10.2196/15283 (PMC7013628; doi:10.2196/15283)
Supplement: Multimedia Appendix 2 [file resprot_v9i1e15283_app2.docx]

**Variables measured by means of the baseline and the closing survey of the FoodTrack study**

| **Baseline survey** | | | |
| --- | --- | --- | --- |
| **Construct** | **# items** | **Outline/ exemplar item** | **Response format** |
| Sex | **1** | What is your sex? | Multiple choice |
| Age | **1** | What is your age? | Open answer |
| Nationality | **1** | What is your nationality | Multiple choice |
| Marital status | **1** | What is your marital status? | Multiple choice |
| Number of household members | **1** | What is the size of your household? | Open answer |
| Household composition | **1** | What is the composition of your household (e.g., 2-person household)? | Multiple choice |
| Highest obtained educational level | **2** | Educational level was based on the highest qualification attained and was classified as: low (less than secondary school or an A-level certificate), medium (A-levels or Dutch A-level equivalent (VWO) graduation certificate), or high (polytechnic or university degree). | Multiple choice |
| Employee status of participant and partner | **2** | Employment status was assessed on working situation (paid work: full time/part time/on call, unemployed/looking for a job, househusband/ wife, disabled and unable to work, other). | Multiple choice |
| Work flexibility | **2** | Are you able to work one or more days from home? | Multiple choice |
| Income level | **1** | What is your net household income? – scale of 500 euros | Multiple choice |
| Financial constraint | **2** | Did you have financial problems last year (e.g., problems paying bills or rent, or buying food)? | Multiple choice |
| Stress [1] | **4** | In the last month, how often have you felt that you were unable to control the important things in your life? | Multiple choice |
| Time stress [2] | **9** | How often do you feel that you never seem to have enough time to get everything done? | Multiple choice |
| Self-control: Brief Self-Control Scale ([3] | **13** | I have a hard time breaking bad habits. | Multiple choice |
| Mastery [4] | **7** | I have little control over the things that happen to me. | Multiple choice |
| FFQ – [5] fruits, vegetables, sugary drinks, snacks | **10** | How many servings of fruit do you normally consume each day? | Multiple choice |

| **Closing survey** | | | |
| --- | --- | --- | --- |
| **Construct** | **# items** | **Outline/exemplar item** | **Response format** |
| Evaluation of app (easy, fun, easiness, effort, completeness of database) | **5** | Was the app easy to use? | 5-point Likert scale |
| Use of GPS tracking | **4** | Was your location tracking “on” during the study period? | Multiple choice |
| Reminder messages | **2** | Were the messages reminding you to enter purchases helpful? | Multiple choice |
| Flat battery | **2** | Did your smartphone have a flat battery during the 7-day period? | Multiple choice |
| Compliance with entering purchases | **5** | Did you enter all the groceries you bought during the 7-day period? | Multiple choice |
| Compliance with entering consumption | **4** | Did you enter all the snacks you ate during the 3-day period? | Multiple choice / 5-point Likert scale |
| Manual entering of portion size | **2** | Could you accurately/easily enter the portion size? | 5-point Likert scale |
| Self-perceived food literacy [6] | **32** | Are you able to see, smell, or feel the quality of fresh foods?  For example of meat, fish, or fruit? | 5-point Likert scale |
| Barriers to healthy eating [7] | **4** | Healthy eating just takes too much time | 5-point Likert scale |
| Prioritizing healthy eating | **4** | There are more important things in life than eating healthily | 5-point Likert scale |
| Food choice motives [8] | **13** | In find it important that the foods I usually eat are nutritious | 5-point Likert scale |

**References**

1. Cohen S, Kamarck T, Mermelstein R. A Global Measure of Perceived Stress. J Health Soc Behav. 1983;24:385. doi:10.2307/2136404.

2. Roxburgh S. “There Just Aren’t Enough Hours in the Day’: The Mental Health Consequences of Time Pressure. J Health Soc Behav. 2004;45:115–31. doi:10.1177/002214650404500201.

3. Tangey JP, Baumeister RF, Boone AL. High Self-Control Predicts Good Adjustment, Less Pathology, Better Grades, and Interpersonal Success. J Pers. 2004;72:271–324. doi:10.1111/j.0022-3506.2004.00263.x.

4. Pearlin LI, Schooler C. The Structure of Coping. 1978. https://www.jstor.org/stable/2136319. Accessed 8 Mar 2019.

5. Willett W. Nutritional epidemiology. Oxford University Press; 1998. http://library.wur.nl/WebQuery/wda/960379. Accessed 17 Apr 2018.

6. Poelman MP, Dijkstra SC, Sponselee H, Kamphuis CBM, Battjes-Fries MCE, Gillebaart M, et al. Towards the measurement of food literacy with respect to healthy eating: the development and validation of the self-perceived food literacy scale among an adult sample in the Netherlands. Int J Behav Nutr Phys Act. 2018;15:54. doi:10.1186/s12966-018-0687-z.

7. Pinho MGM, Mackenbach JD, Charreire H, Oppert J-M, Bárdos H, Glonti K, et al. Exploring the relationship between perceived barriers to healthy eating and dietary behaviours in European adults. Eur J Nutr. 2018;57:1761–70. doi:10.1007/s00394-017-1458-3.

8. Steptoe A, Pollard TM, Wardle J. Development of a Measure of the Motives Underlying the Selection of Food: the Food Choice Questionnaire. 1995. http://www.psychwiki.com/dms/other/labgroup/Measufsdfsdbger345resWeek1/Lindsay/steptoe1995.pdf. Accessed 8 Mar 2019.
